# Presidential Symposium: Stories of Struggle and Resilience: Expert Interdisciplinary Reflections of the Past Year

**DOI:** 10.1093/geroni/igab046.1607

**Published:** 2021-12-17

**Authors:** Danielle Waldron, Kalisha Bonds Johnson

## Abstract

Are you an ESPO member curious about what the “new normal” means for your future career in the field of aging? Or are you a GSA member interested in hearing from your colleagues about their experiences over the past year? Welcome to the ESPO Presidential Symposium! During this session, speakers will share honest and candid insights about their careers in the field of aging amidst the pandemic, racial discrimination/social unrest, and economic insecurity. Speakers in the ESPO Presidential symposium include: Dr. Thomas K.M Cudjoe, Dr. Candace S. Brown, and Dr. Marnin J. Heisel. Dr. Cudjoe, a physician, will discuss his clinical experience treating older adults with COVID-19, the shift to tele-health, and his research on the impact of social isolation on older adults. Dr. Brown, an academician, will discuss how the new attention to the longstanding issues of social injustice in the U.S. shaped her teaching pedagogy, research, student mentorship, and provide critical context regarding the impact of COVID-19 on Black and Indigenous People of Color (BIPOC) professors. Dr. Heisel, a clinical psychologist, will share how his intervention research on resiliency and well-being in older adulthood shifted amidst the “new normal,” as well as how older adults in his clinical practice encountered and coped with difficulties over this past year. As our society confronts social injustice, tackles health implications of COVID-19, and adjusts to a new way of life, we must consider how these factors, together, inform the interdisciplinary stories of struggle and resilience in the field of aging.

